# Continuous Glucose Monitoring in Preterm Infants: The Role of Nutritional Management in Minimizing Glycemic Variability

**DOI:** 10.3390/antiox11101945

**Published:** 2022-09-29

**Authors:** Valeria Musso, Isabella Panfoli, Marcella Battaglini, Giorgia Brigati, Diego Minghetti, Chiara Andreato, Luca A. Ramenghi

**Affiliations:** 1 Pediatric and Neonatology Unit, Istituto Giannina Gaslini, 17100 Savona, Italy; 2 Department of Pharmacy (DIFAR), University of Genoa, 16132 Genova, Italy; 3 Department of Neurosciences, Rehabilitation, Ophthalmology, Genetics, Maternal and Child Health (DINOGMI), University of Genoa, 16132 Genova, Italy; 4 Neonatal Intensive Care Unit, Department Mother and Child, IRCCS Istituto Giannina Gaslini, 16147 Genova, Italy

**Keywords:** continuous glucose monitoring, glycemic variability, enteral feeding, preterm infants

## Abstract

Glycemic variability (GV) is common in preterm infants. In the premature population, GV is a risk factor for morbidity and mortality. Both hypo- and hyperglycemia can impair neurodevelopment. We investigated the impact of continuous versus intermittent tube enteral feeding on GV. In our prospective observational study, 20 preterm infants with a gestational age ≤ 34 weeks at either continuous or intermittent bolus full enteral feeding. For five days, continuous glucose monitoring (CGM) was utilized, which was achieved through the subcutaneous insertion of a sensor. A total of 27,532 measurements of blood glucose were taken. The mean amplitude of glycemic excursions did not differ between the two cohorts statistically. Continuous feeding resulted in higher positive values, increasing the risk of hypo- and hyperglycemia. Subjects who were small for their gestational age had a higher standard deviation during continuous feeding (*p* = 0.001). Data suggest that intermittent bolus nutrition is better for glycemic control than continuous nutrition. Nutritional management optimization of preterm infants appears to be critical for long-term health. In the future, CGM may provide a better understanding of the optimal glucose targets for various clinical conditions, allowing for a more personalized approach to management.

## 1. Introduction

Hypoglycemia is a common metabolic disturbance in newborns caused by an imbalance in the control of glucose metabolism [1,2]. Neonatal hypoglycemia is defined as a blood glucose concentration of less than 47 mg/dL (2.6 mmol/L). According to the American Academy of Pediatrics, asymptomatic blood glucose concentrations of less than 25 mg/dL within 2 h of birth in healthy term neonates do not require treatment, as this is part of a metabolic adaptation to extrauterine life. Intravenous glucose administration is only recommended if blood glucose levels fall below 25 mg/dL within the first 4 h, or below 35 mg/dL (2.0 mmol/L) between 4 and 24 h after birth [3]. In term newborns, the estimated incidence of symptomatic hypoglycemia is 1–3/1000 live births. Preterm infants, on the other hand, have a threefold higher incidence of symptomatic hypoglycemia [4], known to be associated with cerebral damage [5].

Preterm infant hypoglycemia is caused by low glucose-6-phosphatase activity, immature regulatory systems, insufficient glycogen stores, and a limited capacity to access alternative energy sources. Large-for-gestational-age (LGA) infants (47% prevalence), small-for-gestational-age (SGA) infants (52%), and preterm infants (54%), particularly those with a very low birth weight (VLBW) or extremely low birth weight (ELBW), and newborns of diabetic mothers (48%) are more likely to develop hypoglycemia [5,6]. The Pediatric Endocrine Society recommends keeping glucose levels above 50 (2.8 mmol/L) or 60 mg/dL (3.3 mmol/L) [7]. Nonetheless, there is lack of evidence regarding long-term outcomes following neonatal hypoglycemia, as well as evidence of the optimal blood glucose threshold for the management of hypoglycemia [5].

Hyperglycemia, defined as blood glucose levels greater than 8.3 mmol/L (150 mg/dL), is also common in extremely preterm infants (<28 weeks’ gestational age, GA), with an incidence of 450/1000 compared to 20/1000 in term infants [8]. Hyperglycemia is common in VLBW infants during the first few days after birth [9]. It can occur as a result of sepsis and the subsequent perinatal hypoxia and hypovolemia [10]. Neonatal hyperglycemia is usually asymptomatic, though dehydration from osmotic diuresis, weight loss, fever, glucosuria, and metabolic acidosis can occur [8,11]. Risk factors for hyperglycemia in premature newborns include excessive parenteral glucose infusion rates in addiction to endogenous rates of glucose production (4–7 mg/kg/min), low-insulin-dependent tissues (fat and muscle), and a limited insulin response to glucose [12]. Stress-related hormones such as epinephrine and norepinephrine, as well as infused dobutamine and dopamine, can all inhibit insulin secretion [13]. Thresholds for intervention remain controversial, but the most recent ESPGHAN guidelines advise against glucose levels higher than 8 mmol/L (145 mg/dL) [14]. Once potentially treatable causes have been ruled out, hyperglycemia is treated by lowering the glucose infusion rate and administering insulin, though the latter increases the risk of hypoglycemia. Increased amino acids and lipids in parenteral nutrition, as well as the early start of enteral feeding, reduce the risk of hyperglycemia. Moreover, high blood glucose levels increase serum osmolarity, which causes brain cell dehydration and capillary dilation, increasing the risk of cerebral hemorrhage.

Glucose, the primary energy substrate of the brain, accounts for up to 90% of glucose consumption in infants. Glucose metabolism is characterized by constant fluctuations that are influenced by a variety of factors. Glycemic variability (GV) has received a lot of attention in recent years [15]. GV takes into account both intraday and interday variability. In both adult and pediatric intensive care patients, higher GV is an independent risk factor for morbidity and mortality [16,17]. It has also been linked to oxidative stress, which is involved in the development of prematurity complications. Oxidative stress, as well as prematurity complications, causes an increase in GV [18].

Although glycemic values in preterm newborns are closely monitored, the optimal glycemic target has not yet been defined, and there is no consensus on a safe glycemic value for preterm newborns. Despite mounting evidence that suboptimal glycemic homeostasis has a negative impact on psychomotor development, the current standard of care in glycemic monitoring of preterm infants includes measuring capillary or venous glycemic values at least eight times in the first 24 h of life and at least twice in the following days if values are within the normal range. Moreover, there are no recommendations for continuous glycemic monitoring (CGM) in infants. CGM systems are being used successfully in patients with diabetes mellitus, with some even being approved for use in preterm infants. CGM measures interstitial glucose concentration using a fine amperometric glucose oxidase needle sensor inserted into the subcutaneous tissue. Moreover, CGM allows for the detection of asymptomatic hypoglycemia and hyperglycemia, which may benefit from early treatment [19]. CGM has been shown to be useful in targeting glucose control in preterm infants, as well as reliable across all blood glucose ranges [20]. Furthermore, CGM insertion appears to be less painful than capillary sampling [21].

Nutritional preterm management must be optimized for long-term health. Because of the risks associated with vascular catheterization, enteral nutrition (EN) is clearly preferred to total parenteral nutrition when tolerated. Moreover, enteral feeding in the first days of life promotes endocrine adaptation and the maturation of motility patterns, provides luminal nutrients, and may benefit immune function. Recently, early minimal EN administration, at volumes of 4–5 mL/kg/day in the first 7 days of life, has been associated with a higher rate of survival in preterm newborns [22]. Clinical benefits include earlier tolerance of enteral feeds, reduced risk of infection, and earlier discharge [23]. Infants only start to coordinate sucking, swallowing, and breathing around 32 weeks of age or at a body weight of 1500 g, so tube feeding is required for the majority of these infants to ensure sufficient feeding tolerance, support optimal growth, and reduce the risk of aspiration. Therefore, feeding formula or human milk through an orogastric or nasogastric tube is common practice for these infants. The delivery method is either continuous or intermittent bolus. Continuous feeding is defined as delivering EN at a constant speed for 24 h through the use of a nutritional pump [24]. Intermittent bolus feeding is defined as delivering EN multiple times, typically every 2–3 h over 10–20 min via gravity or an electric pump. Due to small sample sizes and methodological limitations, a Cochrane review [25] comparing the clinical effects of continuous versus intermittent nasogastric bolus milk feeding in VLBW infants concluded that the evidence is insufficient for determining an optimal feeding strategy. The effect of continuous versus intermittent enteral feeding on low-birth-weight infants has been studied [26,27], but no consensus has been reached. Intermittent bolus feeding is thought to be more physiologic because it promotes cyclical surges of gastrin, gastric inhibitory peptide, and insulin, and thus gastrointestinal tract development [28]. However, intermittent bolus milk feeding may cause feeding intolerance in premature infants because the absorption capacity of the gastrointestinal tract is easily exceeded. It has been reported that continuous nasogastric feeding improves energy efficiency, reduces feeding intolerance, and improves nutrient absorption, duodenal motor function, splanchnic oxygenation, and growth [29,30].

The purpose of this study was to compare the effectiveness of continuous versus intermittent feeding in terms of maintaining euglycemia in patients receiving total EN, as well as to evaluate blood sugar level stability in SGA infants receiving continuous versus intermittent feeding. Data suggest that intermittent nutrition with bolus feeding is more effective in limiting GV than continuous feeding. This finding was also replicated in the population of SGA babies, who are at a higher risk of hypoglycemia.

## 2. Materials and Methods

### 2.1. Study Design and Population

This is a single-center prospective observational cohort study performed starting from 1 March to August 2021. We prospectively collected, in a specific database, data on nutritional intakes, occurrence of hyperglycemia, hypoglycemia, morbidity, and survival during hospitalization of all newborns with GA ≤ 34 weeks of postmenstrual age on full enteral feeds without any IV glucose supply, as observed in the level-3 neonatal intensive care unit (NICU) of Giannina Gaslini Hospital (Genoa, Italy). Newborns with congenital major malformations, congenital heart disease, gastrointestinal anomalies, contraindications for enteral feeding as determined by treating clinicians, and prior treatment with drugs that can interfere with glycemia were excluded.

### 2.2. Continuous Glucose Measurement (CGM)

Although there are no CGM devices specifically designed for neonatal use, with the size of these devices reduced, their use in preterm infants has become feasible, even in recent trials. We inserted the sensor into the subcutaneous tissue of the lateral thigh, covering it with an occlusive dressing, as is standard practice. In the absence of IV glucose supply, all neonates on full enteral feeding (defined as 130–150 mL/kg/day) received CGM for five days, through the application of the Medtronic Enlite sensor on the outer surface of the thigh after adequate disinfection. The Enlite glucose sensor was used for all patients, and was inserted using the Enlite Serter inserter and used in conjunction with the Medtronic Diabetes glucose measurement system (Medtronic MiniMed MMT-7745WW iPro2), which was used retrospectively. Every 5 min, 24 h a day, the sensor detects interstitial fluid blood glucose and stores it in the device, providing 288 interstitial glucose values per day. The sensor (Enlite sensor) is minimally invasive, soft, flexible, and disposable and is placed subcutaneously. This device is about 1 mm-wide and 10 mm-long, and it is mounted through a hollow needle. It has a platinum electrode that catalyzes interstitial glucose oxidation and generates an electrical current every 10 s. The sensor is connected to a wireless transmitter, which powers the glucose sensor, collects data, and stores it in the device. After completing 5 days of CGM, the neonatologist removed the sensor. In the event of detachment or malfunction, the device was replaced only once. If the patient needed to be transferred to another unit or hospital, the system was removed. The sensors were well-tolerated by our neonates, and no complications occurred. The data were downloaded onto a Windows-based notebook computer running Medtronic Diabetes software. Errors in CGM measurement are caused by three major factors: zero-mean error, drift, and diffusion time lag. Zero-mean error is the random error of the sensor caused by the technology and the interstitial location. Drift is caused by shifts in sensor output between calibration points due to either corrosion or biofilm on the needle surface. The diffusion of glucose into the interstitial compartments causes a time lag; a positive or negative error can be observed when glucose concentration falls or rises, respectively. As per the manufacturer’s instructions, the CGM system must be calibrated with blood glucose values three times a day to maintain its accuracy. Moreover, we used capillary blood glucose values measured by a FreeStyle Optium Neo H glucometer (Abbott Healthcare, Chicago, MA, USA).

### 2.3. Outcome Measurements

According to the Pediatrics Endocrine Society, we defined low-tissue-glucose-value episodes as an interstitial sensor glucose value ≤ 60 mg/dL [7]. Interstitial sensor glucose values >150 mg/dL were used to define high-tissue-glucose-value episodes. This is the most frequently used glucose cutoff in most publications, with >150 mg/dL being consistent with the renal glucose threshold and the observation of glucosuria in the preterm neonate, as well as the commonly used value to define hyperglycemia in older children and adults [9].

### 2.4. Statistical Analysis

Quantitative variables were described as mean and standard deviation if they were normally distributed, and as a median and interquartile range if they were not. The normality of variables was visually assessed by creating relevant histograms. For quantitative variables, the Student’s *t* test was used, as well as Mann–Whitney’s U test for nonnormal variables. Qualitative variables were described as absolute frequencies and percentages. For these variables, comparisons between groups of interest were performed using Pearson’s chi-square test or Fisher’s exact test in the presence of expected values of less than 5 in at least one cell of the corresponding contingency table. Finally, the F test for the ratio of two variances was used to compare the variability of blood glucose measurements in the groups of interest. All statistical analyses were carried out using the STATA statistical package for Windows, version 13.1, with a *p*-value < 0.05 considered statistically significant. GV was measured utilizing EasyGV software, which is available free for noncommercial use at www.phc.ox.ac.uk/research/technologyoutputs/easygv website, accessed on 1 March 2021. EasyGV calculates several measures of GV from CGM data: we reported standard deviation (SD), mean amplitude of glycemic excursions (MAGE), and mean absolute glucose.

## 3. Results

### 3.1. Neonatal Characteristics

During the selected period, a total of 29 patients who met the inclusion criteria were selected, with 10 patients on continuous feeding and 19 patients on intermittent feeding. For the study, 10 of 19 patients in intermittent feeding were selected to match the 10 patients in the continuous feeding group, for a total of 20 enrolled neonates. Neonatal characteristics (Table 1) were similar in the two groups. The study included 9 females and 11 males with an average GA of 31.1 weeks and an average weight of 1.49 kg. Two infants were born small-for-gestational-age (SGA), and four were born large-for-gestational-age (LGA), with the rest were born at the appropriate GA (AGA). During the observation period, only three patients needed respiratory support. No medications that could interfere with glucose metabolism were given to the infants. At admission, neonatal characteristics were similar in the two groups (Table 1). The average corrected age of attainment of full enteral feeding (FEF), when the glucose sensor was applied, was 34.12 weeks (±1.34), and the average weight at FEF was 1.69 kg (±0.33 kg).

**Figure 1:**
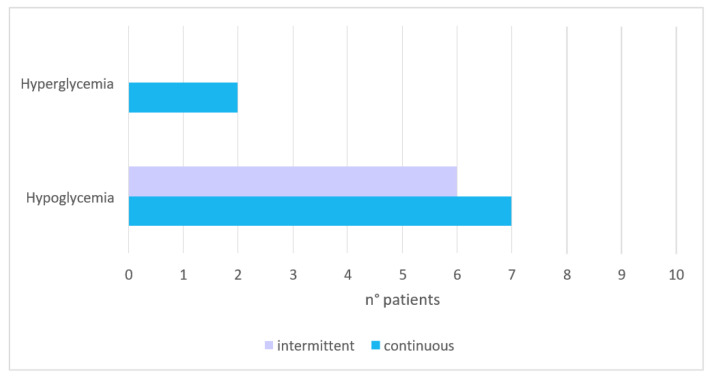
Incidence of hypo- and hyperglycemia based on nutrition strategy.

Detailed clinical information, the mean and SD of blood glucose, the percentage of time spent in hypo- or hyperglycemia, and mean amplitude of glycemic excursions (MAGE) are shown in Supplementary Table S1 for patients on continuous feeding and in Supplementary Table S2 for patients on intermittent feeding. Moreover, seven patients (70%) in the continuous feeding population experienced hypoglycemia, with two (20%) experiencing both hypo- and hyperglycemia. In the intermittent feeding population, six patients (60%) had hypoglycemic episodes, whereas none had hyperglycemia (Figure 1). Six patients on continuous feeding had a positive MAGE, while four patients on intermittent feeding (Figure 1) did not. Although statistical significance was not achieved (according to the nonparametric test, Kruskal–Wallis equality-of-populations rank test, *p* = 0.1356), both median MAGE and SD were lower in the intermittent eating group.

### 3.2. Mean Amplitude Glycemic Excursions

In groups of neonates, we calculated glycemic variability (GV) using MAGE and found no significant differences. As shown in Table 2, the standard deviations of the two groups studied did not differ significantly.

**Figure 2:**
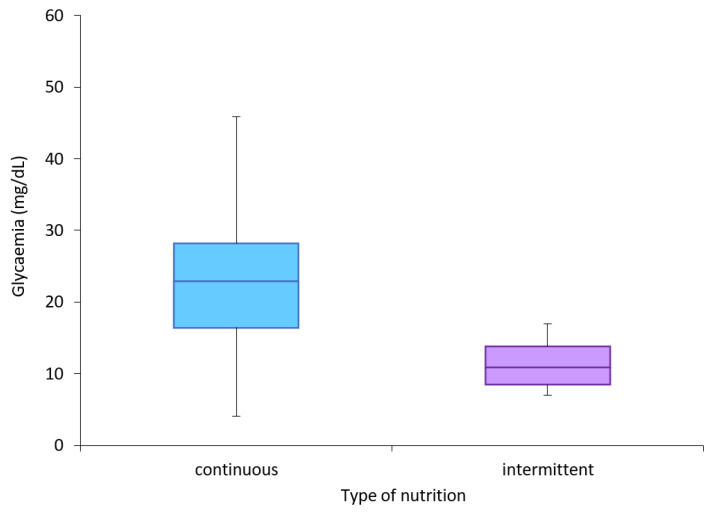
Median, minimum, and maximum MAGE value for each group.

Figure 2 shows that the median amplitude of the wider range was higher in the continuous feeding group. In total, 27,532 measurements of blood glucose were taken in 20 enrolled patients (47.03% from intermittent feeding and 52.97% from continuous feeding).

All glycemic values in both study populations followed a normal distribution, which allowed us to perform statistical calculations.

### 3.3. Association between Nutrition Strategy and Blood Glucose Alterations

**Figure 3:**
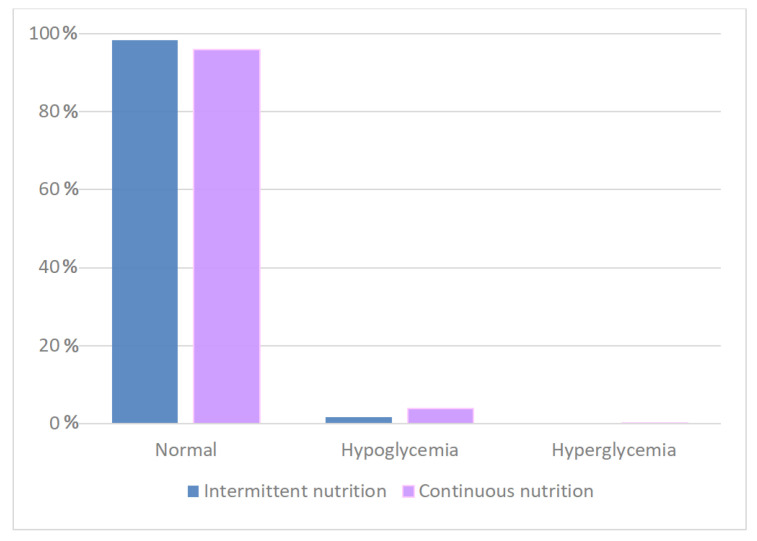
Correlation between glycaemia and type of nutrition: most of the detected blood glucose values were in the reference range. In the continuous feeding group, the incidence of hypoglycemia was higher.

**Figure 4:**
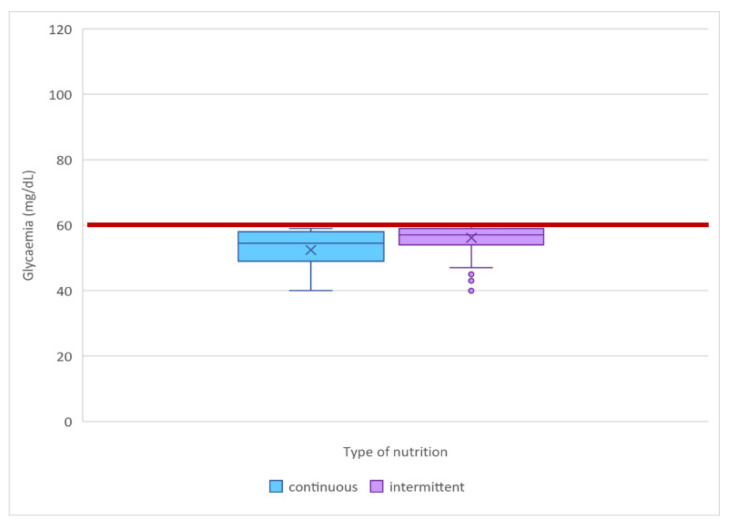
Median, minimum, and maximum hypoglycemic values for each group: in the continuous feeding group, the median was lower, and the amplitude of the wider range was higher. The red line indicates the reference value for euglycemia.

During the CGM days, low-tissue-glucose-value episodes (tissue glucose < 60 mg/dL) were documented in 3.88% of observations in continuous feeding and in 1.6% of observations in intermittent feeding. High-tissue-glucose-value episodes (interstitial glucose values > 150 mg/dL) were observed in 0.11% of measurements in continuous feeding. None of the measurements of the intermittent group was above the reference cutoff with a statistically significant difference (Table 3 and Figure 3). The highest interstitial glucose value was 203 mg/dL in the continuous feeding group and 146 mg/dL in the intermittent group. In the continuous feeding group, low tissue glucose value episodes (tissue glucose < 60 mg/dL) were documented in 3.88% of the observations in continuous feeding versus 1.6% in the intermittent feeding group, and the median of the hypoglycaemic values was lower and the amplitude of the wider range was higher in the former (Figure 4).

### 3.4. Association between Weight for GA and Blood Glucose Alterations Regardless of the Nutrition Strategy

**Figure 5:**
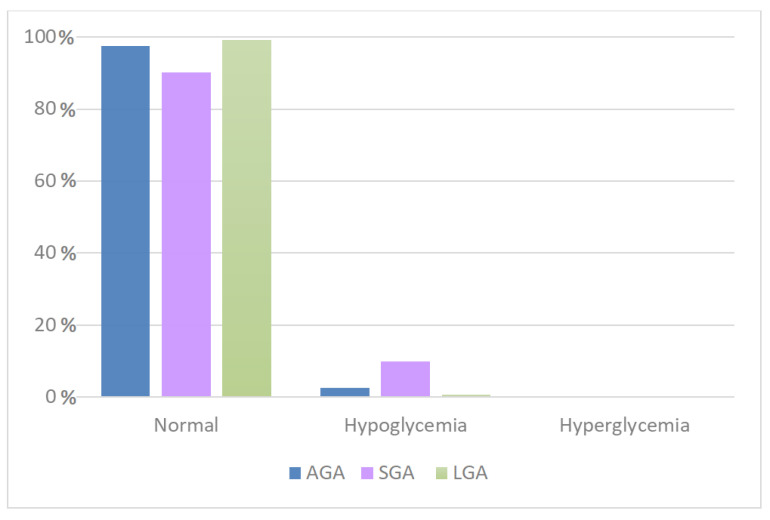
Correlation between glycemia and weight for gestational age. AGA, appropriate for gestational age; SGA, small for gestational age; LGA, large for gestational age.

The majority of the detected blood glucose levels were within the normal range (Figure 5). LGA patients had a lower risk of hypoglycemia than AGA patients (0.71% of total observations) and a mild tendency towards hyperglycemia (0.13%); SGA patients had no episodes of hyperglycemia but a high percentage of hypoglycemic episodes (9.83%) (Table 4). The differences were statistically significant (*p*-value = 0.001).

### 3.5. Association between Weight for GAs and Blood Glucose Alterations in Patient in Intermittent Feeding

**Figure 6:**
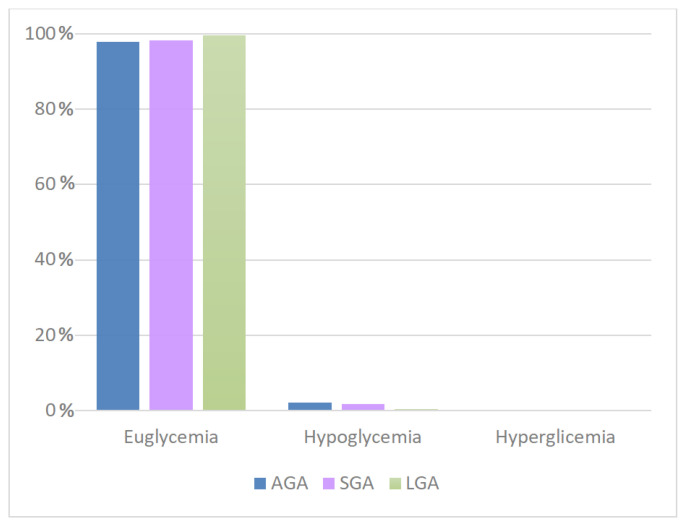
Correlation between glycemia and weight for gestational age in intermittent feeding. AGA, appropriate for gestational age; SGA, small for gestational age; LGA, large for gestational age.

For all categories analyzed, most of the detected blood glucose values were within the reference range; SGA patients had a higher incidence of hypoglycemia than LGA patients (1.68% versus 0.35%); no patients with hyperglycemia were observed in this group (Figure 6). The differences were statistically significant (*p*-value = 0.001, Table 5).

### 3.6. Association between Weight for GAs and Blood Glucose Alterations in Patient in Continuous Feeding

**Figure 7:**
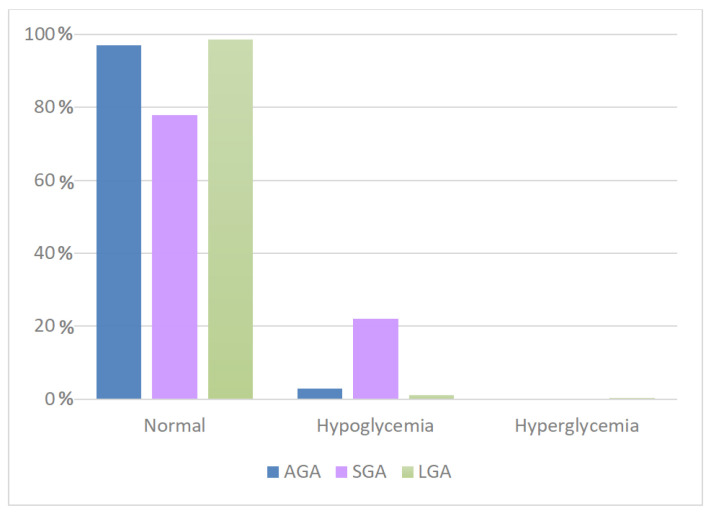
Correlation between glycaemia and weight for gestational age in continuous feeding. AGA, appropriate for gestational age; SGA, small for gestational age; LGA, large for gestational age.

For all categories analyzed, most of the detected blood glucose values were within the reference range. SGA patients had a higher incidence of hypoglycemia (22.15%) (Figure 7), whereas LGA patients had a slight tendency towards hyperglycemia. The differences were statistically significant (Table 6).

### 3.7. Glycemic Variability

The SD was lower in the intermittent group (12.64862 mg/dL) than in the continuous feeding group (14.42761 mg/dL), with a statistically significant difference (*p* < 0.05) (Table 7).

Glycemic observations of SGA patients revealed a statistically greater SD compared to AGA and LGA patients (Table 8).

In the glycemic observations of SGA patients, the standard deviation of continuous feeding was higher (14.31139 mg/dL) than that of intermittent feeding (SD 13.61516 mg/dL), with a statistically significant difference (*p*-value = 0.001) (Table 9). Figure 7 shows how the continuous feeding group had lower median and minimum blood glucose values.

**Figure 8:**
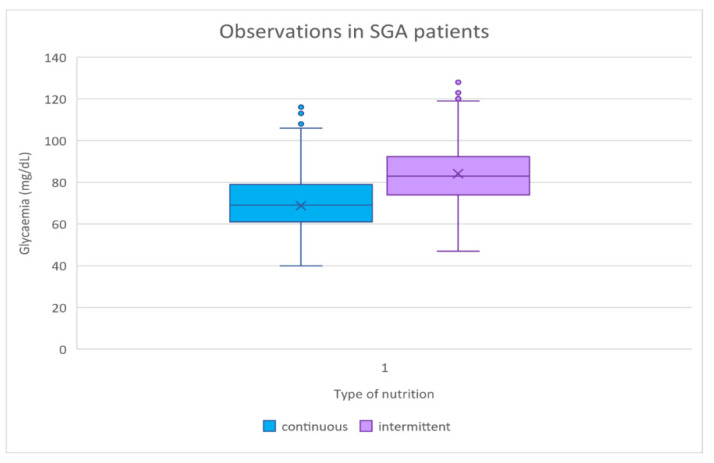
Glycemic observations in SGA patients. SGA, small for gestational age.

**Figure 9:**
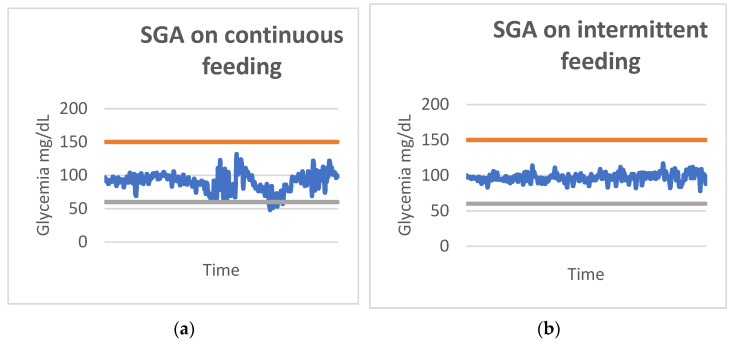
Glycemic trend in SGA for continuous (**a**) and intermittent (**b**) feeding. SGA, small for gestational age. Reference range for lower (grey line) and higher (red line) blood glucose values, are depicted.

Figure 8 depicts how the continuous feeding group had lower median and minimum blood glucose values. The graphs below demonstrate how glycemic control was worse in continuous feeding, with more time spent in hypoglycemia than in intermittent feeding (Figure 9). The color lines represent the reference range for lower and higher blood glucose values, respectively.

## 4. Discussion

For preterm newborns, enteral feeding is the preferred method because it promotes intestinal maturation and neurodevelopment while reducing inflammation [31]. Even though early enteral feeding increases the risk of necrotizing enterocolitis (NEC) and late-onset sepsis, it can be performed in clinically stable VLBW infants. To ensure feeding tolerance and reduce the risk of aspiration, it is always performed using an orogastric or nasogastric tube and nutritional pump [24]. There is little trial evidence to suggest that either method is superior.

The present study sought to assess glycemic variability (GV) in relation to enteral feeding strategy. Data show that, even under stable conditions and FEF, glycemic values fluctuate and appear to be affected by feeding patterns. Even though bolus feeding has been associated with metabolic instability in preterm infants [32], intermittent feeding provided lower GV values than continuous feeding in our cohort. In our population, patients on continuous feeding had a higher GV; more subjects had a positive MAGE (6/10 versus 4/10 in the intermittent group) and at a higher value (mean 25.24 mg/dL) than the other group, where only four patients had a positive mean amplitude of glycemic excursions (MAGE), with a mean value of 11.4 mg/dL. Although the data were not statistically significant (*p*-value of 0.1356 according to the nonparametric test, Kruskal–Wallis equality-of-populations rank test), the mean and SD were lower in the intermittent bolus group than in the other group. Approximately 288 measurements per day per patient were obtained from the continuous monitoring system. A total of 70% (7/10) of patients on continuous feeding had hypoglycemic episodes, compared to 60% (6/10) of patients on intermittent feeding, with a mean duration of 6.17% of the observation period, versus 3% of patients on intermittent bolus feeding. In our study, no patient in the intermittent group had hyperglycemia, which was also rare and mild in the continuous feeding group (2 out of 10 patients with a maximum blood glucose value of 203 mg/dL). However, we found that preterm infants on continuous feeding had a higher rate of prolonged hypoglycemia. During the CGM observations, low-tissue-glucose-value episodes were documented in 3.88% of the observations in continuous feeding and 1.6% of the observations in intermittent feeding, while high-tissue-glucose-value episodes (interstitial glucose values > 150 mg/dL) were observed only in continuous measurement, confirming the hypothesis that the intermittent feeding modality allows for better glycemic control. Notably, intermittent bolus feeding is more physiologic as it promotes cyclical surges of gastrin, gastric inhibitory peptide, and insulin. It also promotes protein synthesis and improves the overall protein balance of the body, thereby promoting gastrointestinal tract development [28]. It has been postulated that intermittent milk feeding may cause food intolerance in premature infants if the absorption capacity of the gastrointestinal tract is exceeded. In our study, no infant had food intolerance.

To the best of our knowledge, this is the first study to look into a possible relationship between GV and the type of nutrition management used in preterm neonates, through CGM. Most studies found no complications with its use [30,33,34]. In our study, continuous monitoring of blood glucose in our cohorts of premature newborns under 34 weeks GA was safe: no local complications (edema, infection, bleeding, or bruising) were observed, and the sensor placement procedure was always well-tolerated when attention was paid to the infant during placement. CGM has several potential advantages. It enables close monitoring of neonates at risk of dysglycemia, reduces the frequency of blood sampling by heel prick [35], and offers a viable alternative to capillary determinations, which cause considerable discomfort to newborns [35,36]. Serial blood glucose monitoring with heel lancing is invasive and has the potential to harm neurodevelopment [37,38]. Moreover, CGM can detect late-onset neonatal metabolic instability.

A limitation is that sensors require a “wetting” phase, which is typically around 2 h, but the time required for stabilization of the signal output has not been specifically studied in neonates [39]. For adequate analysis of normally distributed GV data, arithmetic mean plus or minus SD is commonly used. However, if GV has a positively skewed distribution, some very large values may increase the arithmetic mean and SD, or a lower limit of the reference interval may result in being negative. A previous study confirmed that MAGE and other indexes are related to SD [39]. Therefore, we utilized the MAGE index, which has two main advantages: it does not rely on the mean glucose value and is designed to quantify major glucose swings while excluding minor ones. It can also highlight hyperglycemic fluctuations (MAGE-up, from nadirs to peaks) as well as hypoglycemic fluctuations (MAGE-down, from peaks to nadirs), equivalently weighing hypo- and hyperglycemic fluctuations. In our small-for-gestational-age (SGA) population, intermittent feeding allowed us to maintain a physiological cyclical release of gastrointestinal tract hormones, which aids in the maintenance of euglycemia. SGA neonates have anthropometric values that are less than a certain threshold for infants of the same GA. SGA is caused by maternal, uterine, placental, and fetal factors. In our study, no episodes of hyperglycemia were observed in SGA patients, who instead had a high percentage of hypoglycemic episodes, especially on continuous feeding (9.83% of the total observations), with a statistically significant difference from the other group.

Portable biosensors are revolutionizing the biomedical field. Various methods (such as electrochemical, optical, spectroscopic, and microneedle transdermal techniques) use minimally invasive sources [40]. Despite the development of electrochemical glucose sensors, optical spectroscopic and microneedle transdermal glucose sensing technologies are emerging as promising future alternatives for CGM [40]. Seven optical technologies available for CGM have recently been reviewed [41]. Additionally, a noninvasive sweat glucose sensor based on a wearable hydrogel (Prussian-blue-doped poly(3,4-ethylenedioxythiophene) nanocomposite) patch was recently demonstrated to provide rapid sampling of natural perspiration [42]. CGM improves healthcare by providing a reliable data collection while avoiding the discomfort of the prick test. Noninvasive glucose level monitoring devices are especially used in diabetic patients. CGM is used in children with type 1 diabetes mellitus, but less so in neonatology. A single-center retrospective study reported the use of real-time CGM over a 4-year period in neonates with hypoglycemia [43]. Although CGM has been shown to reduce morbidity in type 1 diabetes pregnancy LGA offsprings, a recent large multicenter observational cohort study on CGM with 300 pregnant women with type 1 diabetes found that neonates were more likely to be hypoglycemic [44].

In some studies, the effects of continuous versus intermittent enteral feeding on low-birth-weight infants were compared using CGM. Continuous feeding was reported to be better than intermittent feeding for gastrointestinal tolerance [27]. By contrast, a study reported that intermittent nasogastric tube feeding in low-birth-weight infants was preferable to semi-continuous feeding [26]. These trials are commonly unblind, which poses a risk of bias.

CGM enabled us to identify hypoglycemic episodes that would not have been detected using capillary determination. There is very little information regarding these episodes, such as which should be treated and whether such treatment would improve outcomes. Routine monitoring is usually only performed on infants in their first weeks of life in the NICU, when they are mostly on parenteral nutrition and glycemic instability is frequent. However, when premature infants achieve complete enteral feeding, metabolic glucose concentration stability is assumed. At this stage, glycemic controls are less frequent, which may underestimate the actual prevalence of hypoglycemic events. It is also possible that identifying episodes of hypoglycemia with CGM leads to overtreatment [45]. Hypoglycemia, which is a common occurrence in premature infants [46], is associated with increased morbidity because it can result in seizures, neurological damage, and visual disturbances, among other effects [6,47]. In newborns, the signs of hypoglycemia are subtle [48]. Neuroglycopenic symptoms include apnea, hypotonia, seizures, lethargy, a weak or high-pitched cry, and coma. Most of these signs are not specific, as they are also manifestations of other neonatal disorders (septicemia, congenital heart disease, ventricular hemorrhage, and respiratory distress syndrome).

Both hypo- and hyperglycemia are linked to potential brain insults and possible neurodevelopmental impairment [35]. Hypoglycemia alters cerebral blood flow, lowers intracellular energy, and disturbs the glutamate metabolism, causing excitotoxicity and ultimately leading to necrosis [9]. A period of rapid brain growth is observed between the 20th and 32nd week of postmenstrual GA, which is characterized by the highest risk of brain damage in the pediatric population [49,50].

There are very limited data on the clinical significance of GV for long-term outcome in preterm infants, as well as its role in the pathogenesis of complications of prematurity [51]. Glucose metabolism undergoes constant fluctuations, whose identification is not easy in premature infants due to a lack of specific symptoms. The clinical significance of large GV regarding future complications of preterm infants remains to be determined [32]. Nonetheless, fluctuations in glucose levels may have a significant impact on long-term outcomes [50].

Rapid blood glucose fluctuations have more specific triggering effects on oxidative stress than chronic sustained hyperglycemia alone [52]. Oxidative stress and reactive oxygen species (ROS) production are known to be involved in brain damage; however, they also appear to be involved in hypoglycemic damage [4]. Increased GV has also been linked to oxidative stress, which is an imbalance between oxidants and antioxidants that is crucial in the development of multiple complications of prematurity [53]. Due to the high energy demand, the transition from a low-oxygen to a higher-oxygen environment and the immaturity of antioxidant systems [54], preterm babies are particularly vulnerable to oxidative stress. A study on human umbilical vein endothelial cells exposed to high glucose found apoptosis of endothelial cells due to ROS overproduction [55,56,57,58]. There is no absolute safe lower margin for blood glucose levels in premature infants as individual susceptibility, cerebral blood flow, rates of glucose uptake, and availability of alternative substrates could individually determine poor neurodevelopmental outcome [6].

## 5. Conclusions

The current data suggest that intermittent nutrition with bolus feeding is more effective than the continuous feeding pattern. This was also observed in the SGA population, which is known to be at higher risk of hypoglycemia. The major limitation of our study is the limited number of enrolled patients; further studies with a higher number of patients are needed to obtain adequate statistical power. The strength of our work is its being an observational study, so glycemic values are not affected by any clinical intervention.

## Figures and Tables

**Table 1:** Neonatal characteristics and duration of hypoglycemia based on CGM. AGA, appropriate for gestational age; SGA, small for gestational age; LGA, large for gestational age; GA, gestational age; FEF, full enteral feeding; NS, nonsignificant.

Infant Characteristics	Continuous Nutrition (*n* = 10)	Intermittent Nutrition (*n* = 10)	*p*-Value
AGA	7	7	NS
SGA	1	1	NS
LGA	2	2	NS
GA (weeks) at FEF	33.5 ± 1.49 (32–36.4)	34.67 ± 0.95 (33.56–35.7)	0.98
Weight at FEF	1.59 ± 0.3 (1.23–2.17)	1.79 ± 0.34 (1.25–2.54)	0.17

**Table 2:** Comparison of MAGE in the intermittent versus continuous feeding group. GV was measured by means of EasyGV software. *p* = 0.1798.

Two-Sample *t* Test with Equal Variances					
Type of Nutrition	N°	Mean (mg/dL)	Std. Err. (mg/dL)	Std. Dev. (mg/dL)	[95% Conf. Interval]
Intermittent	4	11.44	2.20	4.41	4.42–18.45
Continuous	6	25.25	7.39	18.09	6.26–44.23

**Table 3:** Association between type of nutrition and blood glucose alterations.

Glycemia		Intermittent	Continuous	Total
**Normal**	Obs	12,741	14,002	26,743
	%	98.4	96.01	97.13
**Hypoglycemia**	Obs	207	566	773
	%	1.6	3.88	2.81
**Hyperglycemia**	Obs	0	16	16
	%	0	0.11	0.06
**Total**	Obs	12,948	14,584	27,532
	%	100	100	100

**Table 4:** Association between weight for gestational ages (GAs) and blood glucose alterations regardless of the type of nutrition. AGA, appropriate for gestational age; SGA, small for gestational age; LGA, large for gestational age.

Glycemia		AGA	SGA	LGA	Total
**Normal**	Obs	18,265	2322	6156	26,743
	%	97.42	90.17	99.16	97.13
**Hypoglycemia**	Obs	476	253	44	773
	%	2.54	9.83	0.71	2.81
**Hyperglycemia**	Obs	8	0	8	16
	%	0.04	0	0.13	0.06
**Total**	Obs	18,749	2575	6208	27,532
	%	100	100	100	100

**Table 5:** Association between weight for GAs and blood glucose alterations in patients in intermittent feeding. AGA, appropriate for gestational age; SGA, small for gestational age; LGA, large for gestational age.

Glycemia		AGA	SGA	LGA	Total
**Normal**	Obs	7805	1524	3412	12,741
	%	97.88	98.32	99.65	98.40
**Hypoglycemia**	Obs	169	26	12	207
	%	2.12	1.68	0.35	1.60
**Total**	Obs	7974	1550	3424	12,948
	%	100	100	100	100

**Table 6:** Association between weight for GAs and blood glucose alterations in patient in continuous feeding. *p*-value = 0.001. AGA, appropriate for gestational age; SGA, small for gestational age; LGA, large for gestational age.

Glycemia		AGA	LGA	SGA	Total
**Normal**	Obs	10,460	2744	798	14,002
	%	97.08	98.56	77.85	96.01
**Hypoglycemia**	Obs	307	32	227	566
	%	2.85	1.15	22.15	3.88
**Hyperglycemia**	Obs	8	8	0	16
	%	0.07	0.29	0	0.11
**Total**	Obs	18,749	2575	1025	14,584
	%	100	100	100	100

**Table 7:** Variance ratio test based on nutrition strategy AGA, appropriate for gestational age; SGA, small for gestational age; LGA, large for gestational age.

Variance Ratio Test					
Weight for GA	Obs	Mean (mg/dL)	Std. Err (mg/dL)	St. Dev. (mg/dL)	[95% Confidence Interval]
AGA	18,749	84.46	0.095	13.02	84.27–84.65
LGA	6208	89.33	0.17	13.06	89.00–89.65 ***p*-value 0.7262**
SGA	2575	78.61	0.31	15.78	77.45–78.67 ***p*-value 0.001**

**Table 8:** Variance ratio test based on weight for gestational age.

Variance Ratio Test					
Type of Nutrition	Obs	Mean (mg/dL)	Std. Err (mg/dL)	St. Dev. (mg/dL)	[95% Confidence Interval]
Intermittent	12,948	85.76	0.11	12.65	85.54–85.98
Continuous	14,584	84.25	0.12	14.43	84.01–84.48 ***p*-value 0.001**

**Table 9:** Variance ratio test of the observations in SGA babies, based on type of nutrition.

Variance Ratio Test					
Type of Nutrition	Obs	Mean (mg/dL)	Std. Err (mg/dL)	St. Dev. (mg/dL)	[95% Confidence Interval]
Intermittent	1025	68.52	0.45	14.31	67.97–69.73
Continuous	1550	84.15	0.35	13.62	83.47–84.83 ***p*-value 0.001**

## Data Availability

Data is contained within the article and supplementary material.

## Supplementary Materials

The following supporting information can be downloaded at: https://www.mdpi.com/article/10.3390/antiox11101945/s1. Table S1. Characteristics of the continuous feeding population, Table S2. Characteristics of the intermittent feeding population.
