# Supplementary material for: Continuous Glucose Monitoring in Preterm Infants: The Role of Nutritional Management in Minimizing Glycemic Variability
Source: Antioxidants (Basel). 2022 Sep 29;11(10):1945. doi: 10.3390/antiox11101945 (PMC9598281; doi:10.3390/antiox11101945)
Supplement: Supplementary file 1 [file antioxidants-11-01945-s001.zip › antioxidants-1899843-supplementary.pdf]

ID n°	Sex	Birth Weight	Centile InesChart	GA	Weight for GA	Breath support	Full feed	Weight FEF	Mean glucose	Glucose SD	% time hypoglycemica (<60 mg/dL)	% time hyperglycemia (>150 mg/dL)	MAGE
		(g)		(weeks)			(weeks)	(g)	(mg/dL)	(mg/dL)	(%)	(%)	(mg/dL)
1	M	1580	95	29.28	LGA	0	32	1845	97.1	5.7	0	0	0
2	F	910	21	29	AGA	0	32.56	1230	95.2	13.16	1.2	0	28.75
3	M	1450	86	29,28	AGA	0	32	1820	84.6	14.5	3.3	0	15.44
4	F	980	31	28.85	AGA	0	32.7	1325	82.1	7.14	0	0	0
5	M	960	60	27	AGA	1	32.84	1350	87.3	17	1.3	0.6	19.33
6	F	1325	42	30.86	AGA	0	33.28	1505	80.6	8.8	0	0	4
7	F	900	92	25.28	LGA	1	34	1685	86.5	15.2	2.8	0.68	57.5
8	M	1635	26	32.86	AGA	0	35.42	1720	76.9	12.7	8.9	0	0
9	M	1650	69	31.14	AGA	0	36.42	2175	78.8	9.96	3.7	0	0
10	F	980	7	31	SGA	0	34.56	1255	68.8	14.3	22	0	26.46

Supplementary table S1

ID n°	Sex	Birth Weight	Centile InesChart	GA	Weight for GA	Breath support	Full feed	Weight FEF	Mean glucose	Glucose SD	% time hypoglycemia (<60 mg/dL)	% time hyperglycemia (>150 mg/dL)	MAGE
		(g)		(Weeks)			(Weeks)	(g)	(mg/dL)	(mg/dL)	(%)	(%)	(mg/dL)
11	F	1540	50	31.7	AGA	0	33.84	1590	82	7.2	0.24	0	0
12	F	2150	91	32.56	LGA	0	33.84	1915	77.6	7.8	0.64	0	0
13	M	1380	19	31.7	AGA	0	34	1780	89.4	10.15	0	0	0
14	F	1900	26	34	AGA	0	35.28	1700	90	13.1	1.68	0	7
15	M	2445	92	33.28	LGA	0	35.28	2540	97.2	10.16	0	0	0
16	F	1210	3	33.28	SGA	0	35.7	1240	84.16	13.6	1.68	0	0
17	F	1780	22	33.84	AGA	0	35.7	1745	79.45	15	9.15	0	17
18	M	1790	75	31.56	AGA	0	33.7	2030	85.46	11	0	0	9
19	M	1470	68	30.28	AGA	1	33.56	1555	94.9	6.19	0	0	0
20	F	1800	23	33.84	AGA	0	35.84	1890	80.22	12.23	4.78	0	12.75

Supplementary table S2
